# Serum macrophage migration inhibition factor for diagnosing endometriosis and its severity: case-control study

**DOI:** 10.1186/s12905-020-01051-0

**Published:** 2020-09-03

**Authors:** Sahar Mohamed Yehya Elbaradie, Mohamed Sobhy Bakry, Almandouh Hussein Bosilah

**Affiliations:** Obstetric and Gynecology Department, Fayoum University, 23 Mohammed Gonemy of mohammed Elmakreef 6th district, nasr city, Cairo, Fayoum Egypt

**Keywords:** Serum macrophage migration inhibition factor, Endometriosis, Laparoscopy, Infertility, Chronic pelvic pain, Diagnosis

## Abstract

**Background:**

Endometriosis is a long-standing progressive disease that affects women of reproductive age. Macrophage migration inhibitory factor (MIF) is one of non-invasive blood biomarker that was detected in sera of endometriotic patients. The present study aimed to determine the accuracy of serum MIF in diagnosing endometriosis in women with infertility and chronic pelvic pain, and correlate its level to the stage of the disease.

**Methods:**

Observational case-control study conducted at Fayoum University hospital from March 2016 till September 2018. Three hundred women candidate for diagnostic laparoscopy for either infertility or gynecologic chronic pelvic pain were included. The study group included patients with symptoms suggestive of endometriosis or chocolate cyst by ultrasound and proved by laparoscopy and histopathology. The control group included other causes of infertility or pelvic pain. All patients undergone either diagnostic or operative laparoscopy, and before laparoscopy blood sampling for quantitative measurement of macrophage migration inhibitory factor (MIF) protein in serum by ELISA technique.

**Results:**

The level of serum MIF was significantly higher in endometriosis group compared to control group (1.75 ± 1.48 pg/ml and 0.51 ± 0.45 pg/ ml, respectively, P = < 0.001), with a progressive increase with advancing stage (stage I, 1.3 ± 1.03 pg/ml, stage II, 1.7 ± 1.57 pg/ml, stage III, 2.1 ± 1.19 pg/ml and in stage IV, 3.2 ± 2.6 pg/ml). Moreover, in patients presented with pain and infertile patients showed significantly higher levels of serum MIF (1.92 ± 1.13 vs 1.21 ± 1.17 and 1.82 ± 1.13 vs 1.32 ± 0.91 respectively with *p*-value < 0.001). ROC curve of serum MIF with a cut off value of 0.85 pg/ml or more achieves a sensitivity of 80.6%, specificity of 83.3%, positive predictive value of 82.9% and negative predictive value of 81.2%.

**Conclusion:**

Serum MIF might be a promising marker not only for noninvasive diagnosis of endometriosis but as a target for detecting severity as well.

## Synopsis

The level of serum macrophage migration inhibitory factor (MIF) is higher in endometriotic patients and is correlated with disease stage, fertility and pain.

## Background

Endometriosis is an estrogen-dependent, complex and mysterious disease, rarely detected after menopause and before menarche [1]. Despite being a benign gynecologic disease, endometriosis is supposed to mainly result from abnormal invasion and survival of endometrial glands and stroma outside the uterine cavity [2, 3]. Clinically it forms three types of gross lesions: superficial peritoneal lesions, endometriomas or ovarian endometriotic cysts and deep infiltrating lesions [4].

Risk factors, etiology, pathogenesis, and symptomatology vary considerably and still could not be explained. Moreover, there is a great debate about the nature of endometriosis as progressive chronic disease [5].

In 2017 Lagana et al. in their study based on understanding of genetic, epigenetic and biological mechanisms that regulate the development and differentiation of the urogenital tract during intrauterine life, proposed a theory that harmonized the pathogenesis of endometriosis. They hypothesized that, alternation and uncontrol within the mesoderm due to a deregulation of genes leads abnormal placing of stem cells with endometrial phenotype during organogenesis and keep them in an inactive slots. Alternation/activation of peritoneal microenvironment by pro-inflammatory cytokines, adhesion molecules, immune cells and extracellular matrix metalloproteinase create the conditions for survival of ectopic endometrial cells by differentiation, adhesion, and proliferation− + −. What triggers such alterations was not clear, that’s why in their later review, they concluded that a single etiopathogenetic model is not sufficient to explain its complex pathobiology [6, 7].

Overall, most investigators agree that immune dysfunctions, genetic predisposition, hormonal factors and environmental toxins in addition to retrograde menstruation are all-important for initiating the aberrant growth of ectopic endometrium [3, 8]. Abnormal immune and inflammatory changes may be responsible for major symptoms of endometriosis and may have a role for endometrial tissue growth in ectopic areas and development of endometriosis [2]. Yang, et al. and more recently Rakhila et al. in their studies concluded that macrophage migration inhibitory factor (MIF), a potent mitogenic factor for human endothelial cells, could be secreted by endometriotic cells to directly or indirectly stimulate cell proliferation [2, 9].

The prevalence of endometriosis is known to be underestimated because laparoscopy, with or without histological verification, being both successful and safe, is considered the gold standard method to confirm the diagnosis [10]. Although, the literature on the diagnostic value, complications and adverse events of a laparoscopy is very limited, it is safely sure to exclude the diagnosis of endometriosis in women with symptoms and signs of the disease with a negative diagnostic laparoscopy [11]. This attributed to the worldwide known reporting of diagnostic delay between onset of symptoms and diagnosis of endometriosis [12].

In an attempt to introduce non-invasive diagnosis of endometriosis, May et al. in their systematic review identified over 200 possible immunological biomarkers, and concluded that none had been clearly shown to be of clinical use [13]. Furthermore, a Cochrane study reviewed one hundred forty-one studies that evaluated one hundred twenty-two blood biomarkers for endometriosis. These biomarkers included hormones, molecules of cell adhesion, microRNAs (miRNA), inflammatory/apoptosis markers, immune system markers, oxidative stress markers, growth factors, angiogenesis factors, and other proteins [14]. Authors’ concluded that there is a proven advantage of a subset of blood biomarkers for both detecting pelvic endometriosis and for differentiating benign ovarian masses from ovarian endometriomas, however, none of these biomarkers have precise accuracy to be used outside a research setting.

Macrophage migration inhibitory factor (MIF), is one of a non-invasive blood biomarkers that was expressed in endometriosis and has been suggested to have a pivotal role in the pathogenesis of endometriosis as well as infertility and pelvic pain [9]. This pro-inflammatory cytokine could, directly and indirectly, foster angiogenesis, activate proliferation of cells, provoke prostaglandin E synthesis (PGE), and stimulate local synthesis of estradiol in stromal cells of endometriotic tissue was supported by recent studies [15–17].

In 2002, Kats et al. documented increased levels of peritoneal fluid MIF in women with endometriosis that was significantly higher in infertile endometriotic women [18]. Later, in 2005, Cao et al. reported that serum MIF was more than 3 folds higher in women suffering from endometriosis than normal control and its highest level was detected in the advanced stage disease (III–IV); clarifying a reasonable link between MIF and the progression of the disease [19]. Further studies also showed markedly elevated levels of MIF in the peripheral blood [20], as well as in the ectopic endometrial tissue [21] of women with endometriosis elucidating a close relationship between MIF and the pathophysiology of endometriosis, especially in those who had pelvic pain and were infertile.

The present study aimed to evaluate the value of serum macrophage migration inhibitory factor in diagnosing endometriosis in women with infertility and chronic pelvic pain and correlate its level with the stage of the disease.

## Methods

### Design

A case-control observational study from March 2016 till September 2018.

### Setting

The study was conducted at Fayoum University Hospital.

### Patients

Women in the childbearing period presenting with either gynecologic chronic pelvic pain (CPP) or infertility indicated and fit for diagnostic laparoscopy were allocated to either groups of the study. The study group included 150 patients with symptoms suggestive of endometriosis or chocolate cyst by ultrasound and proved by laparoscopy and histopathology. The control group included 150 patients with other causes of infertility or pelvic pain. All women must have no hormonal treatment or surgical intervention for the last 3 months. The patients’ cycle history was documented to determine the cycle phase (proliferative or secretory) and include those of the follicular phase of the cycle. Women suffering from autoimmune, degenerative or neoplastic diseases (e.g chronic/ulcerative colitis, diabetes mellitus, rheumatoid arthritis, multiple sclerosis, asthma, suspicion of malignancy), those with a bleeding tendency or active infection or where laparoscopy is contraindicated were excluded from the study.

Each patient was subjected to informed written consent, detailed medical history, thorough physical and gynecological examinations, laboratory routine preoperative investigations and postoperative histopathological evaluation of biopsy samples of endometriotic lesions. Besides, all patients had a preoperative evaluation by transvaginal ultrasonography (TVU) for assessment of uterine size, uterine cavity, endometrial thickness, exclude other pelvic pathology and search for endometriosis, site, size, and surroundings.

### MIF assay

Five milliliters were collected from the antecubital fossa vein on the morning following admission and before laparoscopy. The collected blood samples were allowed to coagulate at room temperature and centrifuged for 10 min at 3500 rpm to separate cellular elements. The serum was decanted, aliquoted, and stored at–80 °C for further assessment.

Quantitative measurement of MIF protein in serum was measured by ELISA technique following the manufacturer’s protocols {Sandwich assay procedure, sensitivity 6 pg/mL; standard curve range: 8.23–6000 pg/mL; coefficient of variations (CVs) of intra-assay: < 10%; CVs of inter-assay: < 12%}.

In brief, this technique uses captured mouse monoclonal anti-human-MIF antibody (R&D Systems, Minneapolis, MN), a rabbit polyclonal antihuman-MIF antibody for detection, alkaline phosphatase-conjugated goat anti-rabbit IgGs (Chemicon International Inc., Temecula, CA), and para-nitrophenyl phosphate as substrate (RAB0360, Sigma-Aldrich,Inc. Mereck, KGa, Darmstadt, Germany). The optical density was measured at 405 nm, and MIF concentrations were extrapolated from a standard curve using recombinant human MIF. Samples in the MIF ELISA assay were run in duplicate.

### Laparoscopy and biopsy

Laparoscopy was performed for all patients and biopsy from study group. Endometriosis was confirmed by visual inspection and subsequent histopathological evaluation of laparoscopic biopsies of suspected lesions in a hundred and fifty participants (study group).

**Fig. 1:**
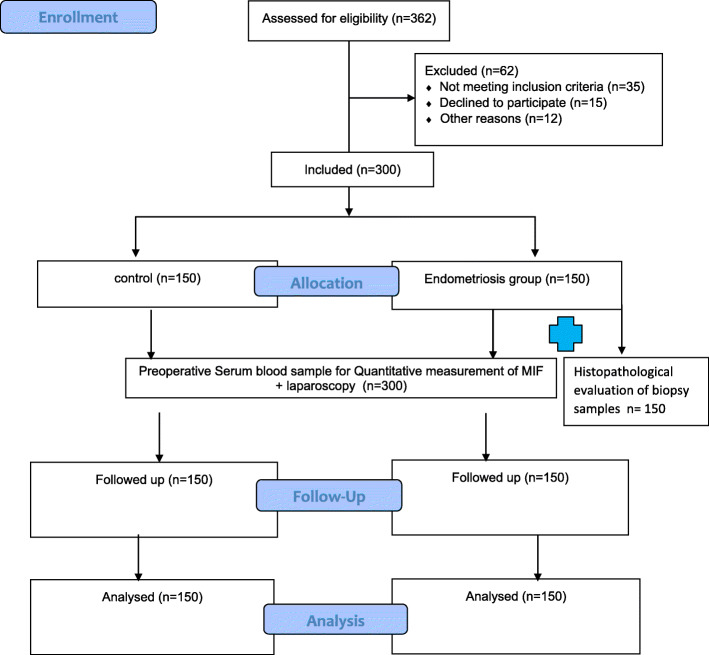
Consort flow chart

The control group, having no visible evidence of endometriosis, included cases of polycystic ovaries, chronic pelvic inflammatory disease, simple ovarian cysts, pelvic adhesions, uterine anomalies, para-ovarian cyst, hydrosalpinx or were laparoscopically free. Endometriosis staging according to the revised American Fertility Society classification (ASRM) point system 1997 was used [22]. Stage I disease (1–5 points) included women with minimal and few superficial implants, stage II (6–15 points) those with mild and more deeper implants, stage III (16–40 points) those with moderate, many deep implants, small cysts on one or both ovaries or presence of filmy adhesions and stage IV disease (> 40 points) included more severe, deep implants, large cysts on one or both ovaries and many dense adhesions. Fig. 1 demonstrates flow chart of the methodology.

### Statistical analysis

Data were statistically described in terms of mean ± standard deviation (± SD), median and range, or frequencies (number of cases) and percentages when appropriate. Comparison of numerical variables between the study groups was done using the Student t-test for independent samples in comparing 2 groups and one-way analysis of variance (ANOVA) test with posthoc multiple 2-group comparisons when comparing more than 2 groups. For comparing categorical data, Chi-square (χ2) test was performed. Accuracy was represented using the terms sensitivity, specificity, positive predictive value, and negative predictive value. Receiver operator characteristic (ROC) analysis was used to determine the optimum cut off value for MIF in diagnosing endometriosis. Two-sided *p* values less than 0.05 were considered statistically significant. All statistical calculations were done using computer program IBM SPSS (Statistical Package for the Social Science; IBM Corp, Armonk, NY, USA) release 22 for Microsoft Windows.

### Sample size

Calculation was done using the comparison of macrophage migration inhibitory factor (MIF) between the 4 stages of endometriosis. As reported in a previous publication [20], the mean ± SD of MIF in stage I-II of endometriosis was approximately 1.36 ± 0.27 ng/ml, while in stages III-IV it was approximately 3.12 ± 0.79 ng/ml. Accordingly, we calculated that the minimum proper sample size was 15 cases in each stage. Since the prevalence of stage IV is approximately 10%, we decided to include 150 cases of endometriosis and 150 control women to be able to reject the null hypothesis with 80% power at α = 0.05 level using Student’s t-test for independent samples. Sample size calculation was done using PS Power and Sample Size Calculations software, version 3.0.11 for MS Windows (William D. Dupont and Walton D., Vanderbilt University, Nashville, Tennessee, USA).

### ClinicalTrials.gov ID trial number

NCT04091997 date of registration 9/13/2019 “Retrospectively registered” URL:https://register.clinicaltrials.gov/prs/app/action/SelectProtocol?sid=S000980H&selectaction=Edit&uid=U0004QXE&ts=3&cx=1jd2jm

## Results

Both groups were comparable regarding age, parity, body mass index, symptoms and type of infertility (Table 1). The endometriosis group included 75 women of stage I, 21 women of stage II, 39 women of stage III and 15 women of stage IV. The control group included 33 women with polycystic ovary disease (PCO), 28 women with pelvic inflammatory disease (PID), 27 women with simple ovarian cyst, 32 women with pelvic adhesions 6 women with uterine anomalies, 2 women with paraovarian cysts, one with hydrosalpinx and 21 women were laparoscopically normal (Table 1).

**Table 1:** Baseline characteristics of endometriotic and non-endometriotic control women

	Endometriosis (***n*** = 150)	Control (n = 150)	***p*** value
Age (years)	28.1 ± 4.2	27.8 ± 3.9	0.552
Parity:			
- P0	72 (48.0%)	69 (46.0%)	0.729
- P1–4	78 (52.0%)	81 (54.0%)	
BMI (kg/m^2^)	26.9 ± 2.7	27.2 ± 2.6	0.328
Symptoms:			
- Pain	114 (76.0%)	105 (70.0%)	0.242
- Infertility	129 (86.0%)	123 (82.0%)	0.345
Type of infertility:			
- Primary	72 (55.8%)	69 (56.1%)	0.964
- Secondary	57 (44.2%)	54 (43.9%)	
Endometriosis stage:			
- Stage I	75 (50.0%)		–
- Stage II	21 (14.0%)		
- Stage III	39 (26.0%)		
- Stage IV	15 (10.0%)		
Lesions in control:			
- PCO		33 (22.0%)	–
- PID		28 (18.7%)	
- Simple ovarian cyst		27 (18.0%)	
- Adhesions		32 (21.3%)	
- Uterine anomalies		6 (4%)	
- Paraovarian cyst		2 (1.3%)	
- hydrosalpinx		1 (0.67%)	
- No lesion		21 (14.0%)	

*BMI* Body mass index, *PCO* Polycystic ovary, *PID* Pelvic inflammatory disease

Women with endometriosis showed more than double-fold increase in serum MIF than non-endometriotic matched women (1.75 ± 1.48 pg/ml vs 0.51 ± 0.45 pg/ml respectively), with a statistically significant difference between groups (*p* <  0.001) (Table 2).

**Table 2:** Comparison of serum macrophage migration inhibitory factor (MIF, pg/ml) between endometriotic patients and those without endometriosis

MIF (pg/ml)	Endometriosis (n = 150)	No-endometriosis (n = 150)	***p***-value
Mean ± SD	1.75 ± 1.48	0.51 ± 0.45	< 0.001
Median (range)	1.6 (0.0–11.1)	0.5 (0.0–3.9)	
95% CI	1.515–1.994	0.439–0.583	

Moreover, the level of serum MIF differed according to the stage of endometriosis with progressive increase with advancing stage (stage I 1.3 ± 1.03 pg/ml-75 patient, stage II 1.7 ± 1.57 pg/ml − 21 patient, stage III 2.1 ± 1.19 pg/ml – 39 patient, and in stage IV 3.2 ± 2.6 pg/ml – 15 patient) (Table 3). Subgroup analysis of serum MIF in different stage disease also showed a statistically significant difference between stage I and stage II, stage II and stage III (*p* > 0.999), stage I and stage IV (*p* <  0.001), but no significant difference between stage II or stage III and stage IV (p 0.009 and 0.057 respectively).

**Table 3:** Comparison of serum macrophage migration inhibitory factor (MIF, pg/ml) between different endometriosis stages

MIF (pg/ml)	Endometriosis Stage				
	Stage I (***n*** = 75)	Stage II (***n*** = 21)	Stage III (***n*** = 39)	Stage IV (n = 15)	***p***-value
Mean ± SD	1.3 ± 1.03	1.7 ± 1.57	2.1 ± 1.19	3.2 ± 2.6	< 0.001
Median (range)	1.2 (0.0–5.4)	1.4 (0.0–6.7)	2.0 (1.0–7.8)	2.2 (1.2–11.1)	
95% CI	1.063–1.537	0.985–2.415	1.714–2.487	1.759–4.641	

As regard symptomatology in endometriotic patients, infertile patients had significantly higher levels of serum MIF compared to fertile endometriotic patients (1.87 ± 1.54 pg/ml vs 1.05 ± 0.74 pg/ml respectively, p <  0.001), with significantly higher levels in those with primary infertility compared to those with secondary infertility (2.3 ± 1.72 pg/ml vs 1.32 ± 1.07 pg/ml respectively, *p* < 0.001). Moreover, there was a statistically significant difference in serum MIF in patients presented with pain in comparison to those without pain (2.02 ± 1.54 pg/ml and 0.871 ± 0.83 pg/ml respectively, *p*-value < 0.001) (Table 4).

**Table 4:** Comparison of serum macrophage migration inhibitory factor (MIF, pg/ml) according to different symptomatology among endometriotic participants

**MIF (pg/ml)**	**Fertile (n = 21)**	**Infertile (*n*** **= 129)**	***p*-value**
Mean ± SD	1.05 ± 0.74	1.87 ± 1.54	0.019 (S)
Median (range)	1.20 (0.0–2.1)	1.60 (0.0–11.1)	
95% CI	0.716–1.389	1.599–2.137	
**MIF (pg/ml)**	**Primary infertility (*n*** **= 72)**	**Secondary infertility (*n*** **= 57)**	**p-value**
Mean ± SD	2.30 ± 1.72	1.32 ± 1.07	< 0.001 (S)
Median (range)	2.20 (0.0–11.1)	1.30 (0.0–7.0)	
95% CI	1.9–2.709	1.034–1.602	
**MIF (pg/ml)**	**No Pain (*n*** **= 36)**	**Pain (*n*** **= 114)**	**p-value**
Mean ± SD	0.871 ± 0.83	2.02 ± 1.54	< 0.001 (S)
Median (range)	0.90 (0.0–2.9)	1.70 (0.0–11.1)	
95% CI	0.587–1.156	1.739–2.307	

(S) significant

**Fig. 2:**
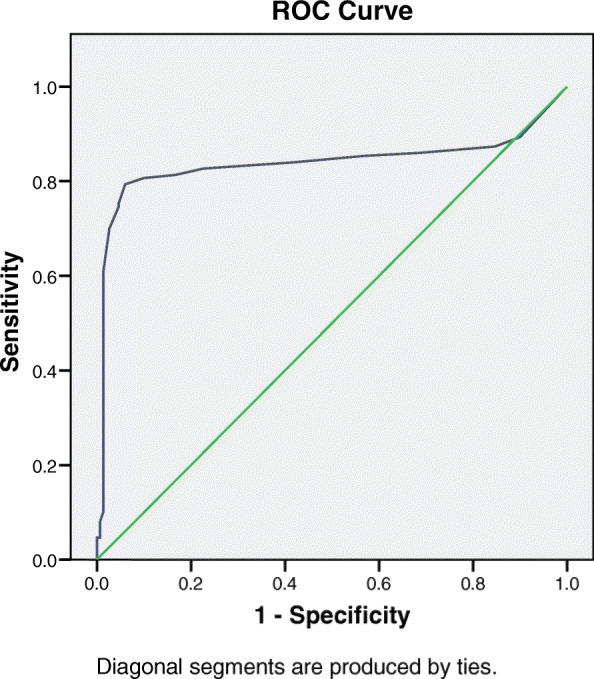
ROC curve

Receiver operating characteristics (ROC) curve was used to define the best cut off value of MIF to diagnose endometriosis, which was 0.85 pg/ml or more. This cutoff achieves a sensitivity of 80.6%, specificity of 83.3%, positive predictive value of 82.9% and negative predictive value of 81.2%. (Fig. 2).

## Discussion

Previous studies reported conflicting results regarding MIF level and mRNA expression across the menstrual cycle. Akoum et al. found a significant decrease of MIF protein in the mid- secretory phase and a significant increase of MIF protein in late secretory and mid-late proliferative phases [21]. Moreover, while Lin et al. and Zhang et al. demonstrated decreased secretory phase serum levels of MIF and MIF expression and increased proliferative phase MIF levels and MIF expression [23, 24], Kats et al. and Arcuri et al found no significant differences [25, 26]. Considering the conflicting results of the previous studies, we included women of the same menstrual cycle phase (immediate postmenstrual proliferative) phase.

In the present study, both endometriotic and control groups were comparable as regard age, parity, and body mass index (BMI). Infertility and the presence of chronic pelvic pain were also comparable. Infertility was recorded in 86 and 82% of patients and control groups respectively while chronic pelvic pain affected 76 and 70% of endometriosis and the control group respectively.

Elevated serum MIF in endometriotic patients compared to control in our study was supported by the study of Morin et al. [20] Serum MIF in their study ranged between 1.3580 ± 0.2690–3.1234 ± 0.7880 pg/ml and in our study, it ranged from 1.3 ± 1.03–3.2 ± 2.6 pg/ml. The diversity of assay techniques, the improvement in the kits used, and the possible higher ongoing inflammatory process could explain these results.

In accordance with previous studies^(21,24)^ the level of serum MIF in our study was much higher in advanced-stage disease than the early-stage disease (1.3 ± 1.03 pg/ml in stage I, 1.7 ± 1.57 pg/ml in stage II, 2.1 ± 1.19 pg/ml in stage III and 3.2 ± 2.6 pg/ml in stage IV) suggesting a relationship with disease progression in the ectopic endometrium. However, our results contradict those of Kate et al., who demonstrated higher levels with active, early-stages and highly vascularized lesions [25]. They reported decreased production of MIF at the mRNA level in more advanced endometriotic lesions supporting a hypothesis that MIF could be a marker of active disease. Also, it contradicts the study of Lin et al. [23] that showed stage-independent MIF expression in ectopic endometrium. Although the explanation for these variations in the level of MIF in respect to the stage of the disease remains unknown and necessitate further investigation, it could be justified by the different numbers and disease stage of patients we studied (75 patients with stage I, 21 with stage II, 39 with stage III and 15 with stage IV) and those selected by Lin et al. (one patient with stage I, six with stage II, 22 with stage III and 11 with stage IV).

In our study, there was a significant difference in serum MIF between fertile endometriotic patients and those with either primary or secondary infertility. Moreover, there was a significant difference between those with primary infertility and those with secondary infertility suggesting a possible effect of MIF on endometrial receptivity. These results are supported by previous studies that detected a significant correlation between infertility and increased MIF levels in endometriotic women [20, 21, 23, 25]. Nevertheless, our results warrant further investigations to elucidate the possible role of MIF in endometriosis-associated infertility and how MIF could affect reproductive function particularly endometrial receptivity.

It is well known that endometriosis-associated pelvic pain is a symptom strongly related to disease existence, but not with disease load [27]. However, our findings supported by previous studies [21, 23, 25] of a significant difference between endometriotic patients with and without pelvic pain could elucidate the possibility that MIF may predict the severity of endometriosis. Though MIF involvement in abnormal bleeding associated with endometriosis, protease secretion induction, and the release of prostaglandins and other pain mediators, as reported in other studies [28, 29] was suggested as a possible role, the mechanisms of MIF’s participation in pain associated with endometriosis, however, remain unclear.

A great strength of our study is the heterogeneity of the control group, including patients who had other pathologies for pain and infertility. This added further reliance on our study is specific to the serum marker tested for diagnosing endometriosis and not just nonspecific symptoms of the disease process of inflammation. Furthermore, the cutoff value of serum MIF in our study of 0.85 pg/ml achieved reliably high sensitivity, specificity, positive and negative predictive values, demonstrating the efficiency of the tested marker in diagnosing patients with endometriosis accurately. In clinical practice, this would probably make the diagnosis and follow up of endometriosis much easier without resorting to surgery and without the need for a panel of assays that was suggested by previous studies [30, 31].

The present study is limited by the inclusion of only postmenstrual women and being a case-control study. Results would be more representative of the targeted population if a relationship between MIF and menstrual cycle phase was proven or refuted and by a cohort design to establish the accuracy of serum MIF to predict endometriosis among symptomatic women and detect the real prevalence of the disease.

## Conclusion

Our study showed a significant increase of serum MIF in endometriotic patients that is correlated with disease stage, pain, and infertility and could be probably a promising marker not only for noninvasive diagnosis of endometriosis but as a target for detecting severity as well.

## Supplementary information

**Additional file 1.**

## Data Availability

The datasets used and/or analyzed during the current study are available from the corresponding author on reasonable request.

## Abbreviations

MIF: Macrophage migration inhibitory factor
ELIZA: Enzyme-linked immunosorbent assay
mRNA: Messenger riboneucliec acid
PGE: Prostaglandin E
CPP: Chronic pelvic pain
TVS: Transvaginal ultrasound
CVs: Coefficient of variations
ASRM: American Society of Reproductive medicine
ROC: Receiver operator characteristic
PCO: Polycystic ovary disease
PID: Pelvic inflammatory disease
BMI: Body mass index
